# Medical Assistance in Dying: A Review of Related Canadian News Media Texts

**DOI:** 10.1007/s10912-022-09764-z

**Published:** 2022-12-01

**Authors:** Julia Brassolotto, Alessandro Manduca-Barone, Paige Zurbrigg

**Affiliations:** Faculty of Health Sciences, University of Lethbridge, 4401 University Drive, Lethbridge, Alberta T1K 3M4 Canada

**Keywords:** Medical assistance in dying (MAiD), Euthanasia, News media, Textual analysis, Canada

## Abstract

Medical assistance in dying (MAiD) was legalized in Canada in 2016. Canadians’ opinions on the service are nuanced, particularly as the legislation changes over time. In this paper, we outline findings from our review of representations of MAiD in Canadian news media texts since its legalization. These stories reflect the concerns, priorities, and experiences of key stakeholders and function pedagogically, shaping public opinion about MAiD. We discuss this review of Canadian news media on MAiD, provide examples of four key themes we identified (vulnerability, autonomy, dignity, and human rights), and discuss their implications for health policy and equity. Though key stakeholders share the values of autonomy, dignity, and human rights, they appeal to them in diverse ways, sometimes with conflicting policy demands. These representations offer a useful gauge of how views about MAiD continue to shift alongside changes in federal legislation. These stories can influence related policies, respond to the powerful voices that shape MAiD legislation, and have the potential to change national conversations. Our analysis adds to the existing body of scholarship on MAiD by examining post-Bill C-7 news media, identifying related health equity issues and tensions, and discussing potential impacts of MAiD’s representations in news media.

## Introduction

Medical assistance in dying, also known as MAiD, was legalized in Canada in 2016. This law permits physicians and nurse practitioners to a) administer a substance that causes death for eligible patients who have requested it or b) prescribe the substance so that patients can self-administer, causing their own death. Government communications and polling often describe MAiD as having popular support with the Canadian public (regularly 75–85 percent support since 2015) (Pennings and Reid 2020). However, a deeper look reveals that Canadians’ opinions about the service are nuanced and complex (Pennings and Reid 2020). MAiD is a healthcare service shrouded in ethical debate with considerable implications for health equity. In the spring of 2021, Bill C-7 was approved and expanded the MAiD eligibility criteria. Specifically, it allows for individuals whose natural death is *not* reasonably foreseeable to access MAiD on the grounds of “grievous and irremediable suffering” resulting from “a serious and incurable illness, disease, or disability” (Government of Canada 2022). This expansion has contributed to both ongoing and emerging public debates about the ethics of assisted dying.

In this paper, we outline findings from our review of representations of MAiD in mainstream Canadian news media texts since the time of its legalization. These stories can function pedagogically, shape public opinion, and reflect the concerns, priorities, and experiences of key stakeholders. Given that news media is a social institution that produces public knowledge (Schudson 1995), it has the power to influence policy, hold power to account, and change national conversations (Van Dijk 1995; Hayes et al. 2007). Additionally, mass media are especially influential in shaping discourses about health and health care (Hayes et al. 2007; Dorfman 2001; Seale 2003).

In what follows, we review Canadian news media on MAiD, provide examples of the four key themes we identified (vulnerability, autonomy, dignity, and human rights), and discuss their implications for health policy and equity. Though key stakeholders share the values of autonomy, dignity, and human rights, they appeal to them in diverse ways, sometimes with conflicting policy demands. Similarly, we identified dissensus about what constitutes vulnerability and how the law ought to protect those who are considered vulnerable.

## Background

### Legislative context

Until 2015, the Supreme Court of Canada upheld a blanket prohibition against assisted death. However, that year, in *Carter v Canada*,^1^ the Supreme Court unanimously voted that the prohibition was unconstitutional. It established an exemption allowing individuals who met the criteria of the Court’s decision to apply for judicial authorization and receive a medically assisted death. This continued until the new MAiD legislation was implemented via Bill C-14 in June 2016. Between 2016 and 2020, over 21,000 Canadians received medically assisted deaths, accounting for 2.5 percent of deaths in 2020 (Government of Canada 2022).

In the fall of 2019, the Superior Court of Québec declared in *Truchon v Canada*^2^ the “reasonable foreseeability of natural death” eligibility criterion unconstitutional. In response, the federal government introduced Bill C-7. This Bill would amend the Criminal Code and allow individuals whose natural deaths are not reasonably foreseeable to apply for MAiD. Bill C-7 received Royal Assent in March 2021.

### Discursive context

Debates about MAiD have shifted over time. Historically, sanctity of life and slippery slope arguments dominated public discourse (Borovoy 1994; Chambers 2014; Somerville 2014). For many years, academic and legal debates focused on the moral distinction between passive and active forms of euthanasia. Passive euthanasia involves withholding or withdrawing life-sustaining treatment while active euthanasia involves the administration of an intervention—for example, injecting a patient with a lethal substance in order to cause death. The distinction between active and passive forms of euthanasia was often characterized as killing versus letting die (Rachels 1975) or act versus omission (Downie 2004). For decades, the prevalent thinking in Western bioethics scholarship was that passive euthanasia was morally acceptable and active forms were morally condemnable; until 2016, the Criminal Code of Canada reflected this reasoning. The American Medical Association’s *Code of Medical Ethics* opinions continue to reflect this distinction (see, AMA 2022). However, multiple scholars have argued that the distinction is not morally relevant (Rachels 1975; Downie 2004). Instead, they contend that moral evaluations likely hinge on a separate aspect, such as the intention behind the act.

Public discourse in Canada has largely shifted away from these philosophical debates (e.g., “should we permit assisted death?”) and is now focused predominantly on policy development (e.g., “how do we provide assisted death?”). This public discourse includes debates surrounding eligibility criteria for MAiD and the related safeguards. The passing of Bill C-7 has heightened controversy, and the bill has received criticism for the extent of its permissiveness. As a result, many disability rights advocates fear that this change will lead to further discrimination against people with disabilities and sends the message to society that their lives are not of equal worth (Lemmens and Krakowitz-Broker 2020; Ackermann and Tindale 2021).

### Media representations of MAiD

Ours is not the first media analysis on this topic. Previous media analyses have centered on the morality and ethics of permitting MAiD. Earlier publications highlighted the ways in which MAiD has been portrayed in various media sources and the influence that discursive choices have on perceptions of related morality and ethics (Wright et al. 2015; Crumley et al. 2019; Street and Kissane 2001; Pollock and Yulis 2004; Kalwinsky 1998). Street and Kissane (2001) highlight discursive representations of the body in Australian media pertaining to MAiD. Kalwinsky (1998) examines how the *New York Times*’ framing of MAiD has shifted over time. Specifically, Kalwinsky (1998) addresses how the shifts in discourses have both reflected and incited changes to how we think about life and death, which then becomes reflected in media. Previous analyses have also examined the portrayal of physician perspectives and attitudes toward MAiD in media texts (Wright et al. 2015; Crumley et al. 2019). For instance, Wright et al. (2015) found that some Canadian physicians’ public statements reveal that they feel that MAiD conflicts with the general mandate of medical practice (i.e., heal and do no harm). Other scholars have explored the ways in which physicians’ roles in the provision of MAiD have been portrayed through media over time (Crumley et al. 2019; Kalwinsky 1998). In addition, Wright et al. (2015) identified “advocacy for existing palliative care options” as a common discourse in news media sources. Some studies also recognize disability rights advocacy in their media analyses (Schwartz and Lutfiyya 2009; Haller and Ralph 2001).

Although previous media analyses examine the topic of MAiD, there is currently a gap in the literature that we seek to address through this review. Most earlier publications address the topic more generally without a specific focus on the Canadian context (Street and Kissane 2001; Kalwinsky 1998; Pollock and Yulis 2004). However, the Canadian context is particularly salient given the ongoing process of legalization of MAiD. Furthermore, the existing Canadian media analyses of MAiD predate recent policy and legislative changes. There have been three media analyses of MAiD within Canada. Two of these studies focus primarily on physician attitudes and perspectives (Wright et al. 2015; Crumley et al. 2019) and focus on: MAiD as a social issue, the legal and professional aspects of physician involvement, and the political aspects of assisted dying. Crumley et al.’s (2019) work, for instance, demonstrates that public opinion polls reported in news media reflected growing support for MAiD over time (e.g., from 44 percent support in 1968 to 77 percent support in 1990) despite physician hesitance or opposition. The third Canadian review also tracked discursive shifts prior to legalization, with attention to overarching frames of legality, social progress, and service provision (Burlone 2020). Notably, there have been no media analyses of MAiD in Canada published since the passage of Bill C-7. Our analysis adds to this body of scholarship by examining news media leading up to and following the introduction of Bill C-7, identifying related health equity issues and tensions, and discussing the potential impacts of MAiD’s representation in news media. Our findings build upon prior media analyses by addressing recent news representations and continuing the discussion of MAiD as a legal, social, and political issue in Canada.

## Methods

This paper provides a textual analysis of MAiD in Canadian digital print news media in the tradition of cultural studies (Rozanova 2010; Wada, Clarke, and Rozanova 2015). Using the ProQuest Canadian Newsstream online database and Google News, each author searched for English-language articles about MAiD published in mainstream news outlets since 2015 (the year in which the Supreme Court of Canada ruled that the prohibition of MAiD was unconstitutional). Since MAiD is legislated at the federal level, we purposively selected articles that were from national news sources (e.g., the Canadian Broadcasting Corporation [CBC], the *National Pos*t, and *The Globe and Mail*) and magazines (e.g., *MacLean’s* and *Policy Options*) as well as regional publications with a national audience (e.g., the *Toronto Star*). We conducted this review from September 2020 to March 2021, during the lead-up to the legalization of Bill C-7. Bill C-7’s amendments have distinct legal, social, and political implications that have received considerable public scrutiny. We used purposive sampling to select news items that highlight related social and political issues. We intentionally selected examples that highlight the dominant discourses about MAiD in Canada during this period (Grigorovich 2020). This included particular attention to equity issues related to disability, poverty, Indigeneity, incarceration, mental illness, and other social locations that would be impacted by C-7’s expanded eligibility criteria. We omitted items that strictly reported on legislative changes (i.e., simply the fact that C-7 was proposed) with limited to no discussion of the politics or potential implications of such changes. We also omitted repeat articles (i.e., pieces that told the same story in multiple outlets).

We consolidated our respective findings and included 32 items in our sample: a combination of columns, opinion pieces, and articles. Table 1 below shows all items analyzed in our media text review. Because we were interested in the key themes about MAiD in Canadian media (rather than a systematic content analysis), we were more concerned with representative, resonant examples than an exhaustive collection of every article published on the topic since 2015. Since MAiD-related policies continue to change following the passage of Bill C-7 in March 2021, we expect ongoing publication of news items about the topic that we will inevitably not be able to include in this review.

**Table 1.** Sources included in media text review

Title	Date	Author(s)	Source
Indigenous parliamentarians brought unique perspective to assisted dying debate	Jul 4, 2016	J. Bryden	*CBC News*
Euthanasia may save $139M, study says; Argues savings dwarf costs of assisted death	Jan 24, 2017	S. Kirkey	*National Post*
‘The solution is assisted life’: Offered death, terminally ill Ont. man files lawsuit	Mar 15, 2018	*CTV News*	*CTV News*
Watchdog calls for ‘compassionate’ parole as prison adopts new assisted death policy	Feb 25, 2018	K. Harris	*CBC News*
Chronically ill man releases audio of hospital staff offering assisted death	Aug 2, 2018	*CTV News*	*CTV News*
Medically assisted dying needs more monitoring	Aug 29, 2018	C. Frazee	*Toronto Star*
Expert panel on MAiD has left us with many questions, but no solutions	Dec 14, 2018	A. Picard	*The Globe and Mail*
Euthanasia’s slippery slope gets steeper in Canada every year	May 25, 2019	L. Corbella	*National Post*
Liberty or equality? Unrestricted access to medically assisted death endangers vulnerable people	Oct 12, 2019	R. Enns	*CBC News*
He wanted to bring attention to assisted death - so he died by suicide in front of Alberta’s legislature	Dec 7, 2019	N. Yousif	*Toronto Star*
Wealthier patients more likely to use medical assistance in dying, data show	Feb 12, 2020	K. Grant	*The Globe and Mail*
Opinion: MAID should not be offered to people with mental illness	Feb 18, 2020	G. Vrakas	*Montreal Gazette*
Why medical assistance in dying must treat mental and physical illness equally	Feb 27, 2020	J. Scully	*CBC News*
MAID advocate receives assisted death early because of coronavirus fears	Apr 9, 2020	T. Thanh Ha	*The Globe and Mail*
The fight over medical assistance in dying in one B.C. community is getting ugly	Jun 6, 2020	G. Mason	*The Globe and Mail*
Rush to expand MAiD turns policy-making on its head	Jul 11, 2020	H. Schipper & H. Swain	*The Vancouver Sun*
How medical assistance in dying law has affected my family: A caregivers perspective	Jul 24, 2020	J. LeBlanc	*CBC News*
ODSP recipients calling for help, exploring assisted dying	Sep 2, 2020	C. Mulligan & M. Yawar	*City News Toronto*
Q and A with Heidi Janz: COVID-19 exposed ableism, assisted death Bill C-7 endangers those with disabilities	Oct 25, 2020	L. Boothby	*Edmonton Journal*
Medical assistance in dying bill an important step forward for Canadians with dementia	Oct 27, 2020	J. Downie	*Policy Options*
Why the federal government should rethink its new medical assistance in dying law	Nov 10, 2020	T. Lemmens & L. Krakowitz-Broker	*CBC News*
Disability advocates say assisted dying bill fails to protect vulnerable Canadians	Nov 10, 2020	K. Harris	*CBC News*
Dying for the right to live	Nov 12, 2020	G. Peters	*MacLean’s*
Compassionate release should be prioritized over MAID in Canadian prisons, says expert	Nov 17, 2020	T. Mahboob	*CBC News*
‘Cold comfort to be offered the choice to die’ when not offered support to live, says disability advocate	Nov 19, 2020	*CBC News*	*CBC News*
Assisted-death bill sends wrong message to Indigenous people, advocates say	Nov 24, 2020	S. Levitz	*National Post*
Canadians’ views on assisted dying are complex	Dec 4, 2020	R. Pennings & A. Reid	*Policy Options*
MAID litigant says disability doesn’t make her vulnerable to pressure to end her life	Dec 16, 2020	J. Bryden	*CTV News*
Proposed changes to assisted-dying law make death a viable option for people with disabilities, advocate fears	Dec 17, 2020	*CBC News*	*CBC News*
Living and dying with dignity: third chance to get assisted dying bill right	Dec 20, 2020	S. Ho, A. Favaro & E. St. Philip	*CTV News*
If medically assisted death becomes more accessible for Canadians, we have a moral obligation to make living well—through housing, mental health supports—accessible too	Feb 11, 2021	N. Dosani	*Toronto Star*
Canada is plunging towards a human rights disaster for disabled people	Feb 19, 2021	H. Braswell	*Washington Post*

We began our analysis with independent careful readings of these articles and re-read them to identify patterns in representation (e.g., the language used to describe prospective patients and/or their motivations) (Rice and Ezzy 1999). We met as a team to discuss and refine key themes. This process allowed us to identify how related issues were framed, which voices or perspectives were included, and what was considered *newsworthy*. As Stuart Hall (1981, 35) has explained, though focused on the topic of race, “[t]he media are not only a powerful source of ideas about race. They are also one place where these ideas are articulated, worked on, transformed, and elaborated.” With this in mind, we worked together to identify and articulate how MAiD representations have developed and transformed in recent years. In what follows, we elaborate on what is included in the key themes, provide examples of their representations, and discuss their implications for health policy and equity.

## Findings

We found a diverse array of perspectives and voices in these articles, both in terms of authors and subjects. This diversity includes pieces written by or showcasing self-identified advocates, activists, members of marginalized or stakeholder groups, a family caregiver, and experts in the field(s). This variety of authorship and perspectives is a noteworthy shift away from the dominant media focus on physician perspectives (Wright et al. 2015; Crumley et al. 2019) or broad Canadian attitudes (Pennings and Reid 2020) depicted in earlier media. There was significant attention paid to marginalized populations, suggesting that this is what is considered especially newsworthy as MAiD legislation evolves. We categorized our findings under four key themes: 1) vulnerability, 2) autonomy, 3) dignity, and 4) human rights.

### Vulnerability

The most popular concept invoked in these media texts was vulnerability. This refers to vulnerability to exploitation or coercion that individuals from marginalized groups may experience in light of social inequities. Forty-eight percent of Canadians identify as “cautious supporters” of MAiD, with “protection of the vulnerable” as their top concern or caveat (Pennings and Reid 2020). The safeguards included in MAiD legislation are indeed intended to “protect vulnerable persons” (Government of Canada 2022). For instance, a person seeking MAiD must meet all medical eligibility criteria, be of age, and have the cognitive capacity to consent to the process; the assessors and witnesses must be independent of one another and not have any conflict of interest; and there are waiting periods between different stages of the process. Furthermore, guidance to health professionals stipulates that when assessing a patient for MAiD eligibility, the care provider ought to ensure that the patient is not seeking MAiD because of unmet needs or lack of access to other resources (Canadian Nurses Association 2017). However, because of systemic injustices and changes to the MAiD eligibility criteria in Bill C-7, there has been increased news media recognition of the limits of the healthcare system to address broader social inequities.

In many of these media texts, MAiD was represented as a potential threat to the lives of marginalized people. Specifically, we found increased attention to the vulnerability of those with disabilities (Enns 2019; Lemmens and Krakowitz-Broker 2020; CBC Radio 2020a, b; Harris 2020; Bryden 2020; Boothby 2020; Peters 2020), Indigenous peoples (Bryden 2016; Levitz 2020), prison inmates (Harris 2018; Mahboob 2020), those living in poverty (Mulligan and Yawar 2020; Peters 2020; Dosani 2021), and those with mental illnesses (Scully 2020; Vrakas 2020; Dosani 2021). Most of these pieces acknowledge that we live in an inequitable world and that social, political, legal, medical, and economic structures are primarily what make people vulnerable.

For instance, several media pieces featured discussion of how Bill C-7 disproportionately negatively affects people with disabilities. Authors explained how C-7 makes disability itself grounds for MAiD eligibility in ways that other marginalized groups do not face (Boothby 2020). In her address to the House of Commons Standing Committee on Justice and Human Rights,^3^ Professor Emeritus and activist Catherine Frazee stated:

Bill C-7 begs the question, why us? Why only us? Why only people whose bodies are altered or painful or in decline? Why not everyone who lives outside the margins of a decent life, everyone who resorts to an overdose, a high bridge, a shotgun carried out into the woods? Why not everyone who decides that their quality of life is in the ditch?

In other news media publications (e.g., Frazee 2018; CBC Radio 2020a), Frazee has highlighted the vulnerability of people with disabilities living in an ableist world. She claims that the changing MAiD legislation can and will shift public perception and lead non-disabled people to see disabled people’s lives as not worth living, rendering them further vulnerable to social and healthcare discrimination. Similarly, Lemmens and Krakowitz-Broker (2020) argue that the C-7 legislation “ignores the challenging context in which people with disabilities and chronic illness must make a decision regarding MAiD.” The new bill does not explicitly require that all reasonable treatments be made available and tried before a patient can access MAiD—a requirement in all other jurisdictions that permit medically assisted deaths (Lemmens and Krakowitz-Broker 2020).

Several articles also highlight the fact that the COVID-19 pandemic has exacerbated the vulnerability of people with disabilities. The pandemic exposed widespread ableism (Boothby 2020), increased social isolation (Lemmens and Krakowitz-Broker 2020), and had negative financial impacts on disabled people that impaired their ability to live well (Mulligan and Yawar 2020). These pieces reveal that changing external conditions can amplify (or ameliorate) vulnerability.

There is, however, some disagreement about representing people with disabilities as vulnerable. Nicole Gladu, who lives with post-polio syndrome, was one of the two Quebec residents who successfully challenged the constitutionality of MAiD only being provided to people whose natural death is reasonably foreseeable (Bryden 2020). She dismissed the reasonably foreseeable death safeguard of Bill C-14, claiming that “vulnerability is a concept used ad nauseum by paternalistic people in good health for standing in the way of MAiD” (Gladu quoted in Bryden 2020). She also rejects the argument that she is a vulnerable person in need of protection under the law, claiming that her disability has not prevented her from living a rich life. Bryden’s article represents vulnerability primarily through the lens of physical disability, without attention to the ways in which other social locations intersect to make an individual more or less vulnerable. Other articles that we reviewed were more attentive to this.

Vulnerability was also invoked in news media texts about MAiD and Indigenous peoples. One article referenced the medical racism that Indigenous people face when receiving health care in Canada, including a history of receiving procedures without their consent (Levitz 2020). There is fear that Indigenous peoples’ lives will be devalued in healthcare settings and concern about protecting vulnerable people from what is seen as an inducement to suicide (Bryden 2016). Colonialism and systemic racism are thus framed as making Indigenous people vulnerable to potential exploitation under MAiD. Furthermore, Bryden (2016) notes that MAiD does not account for Indigenous worldviews about choosing to end one’s life. Presenting MAiD as an option for Indigenous patients is viewed by some as a form of neocolonialism (Bryden 2016). The legalization of MAiD is also described as having negative implications regarding the normalization of suicide (Levitz 2020), particularly when there have been concerted efforts toward suicide prevention in Indigenous communities.

Lastly, there was some news media attention to the vulnerability of people with mental illnesses. People living with mental illnesses were presented as vulnerable because the Federal Government announced in spring 2021 that, after March 2023, people with a mental illness as their sole underlying medical condition will be able to access MAiD so long as they satisfy all other eligibility and assessment criteria (Government of Canada 2022). Because provincial health insurance plans do not typically include mental health services, access to appropriate, affordable, and timely mental health services and treatments is limited in Canada to those who can afford them, either through individual wealth or access to private health care insurance (Vrakas 2020). Since the Canadian MAiD legislation does not explicitly require that the patient has tried and exhausted all available treatments prior to requesting MAiD, there is concern that permitting MAiD on the grounds of mental illness makes these patients vulnerable to exploitation or systemically coercive death (Vrakas 2020; Dosani 2021). One palliative care doctor noted that he can successfully arrange for a medically assisted death for his patients in a more timely and organized process than he can arrange access to mental health care services (Dosani 2021). He argues that with psychosocial supports and social determinants of health addressed, many patients’ quality of life improves, and they may reconsider their requests for MAiD. Once again, vulnerability is depicted as contingent upon broader social structures and service accessibility.

Like the discourse around disability, media coverage reflects dissensus about the vulnerability of people with mental illnesses. Scully (2020) argues that those with mental illnesses are not necessarily vulnerable. He suggests that in MAiD legislation, mental suffering should be treated like physical suffering and that those with mental illnesses will be denied a voice if the service is prohibited for them. Without said access to MAiD, Scully (2020) argues that there is no relief from suffering unless the individual chooses to die by suicide, which can include unsuccessful attempts, brutal methods, and trauma for loved ones.

The disagreement in media discussions about the vulnerability of those with mental illnesses reflects the mixed feelings of the broader Canadian public on this topic: “Almost seven in 10 Canadians say policy-makers should give considerable weight to the concern that expanding MAiD may lead to people with mental-health issues like depression choosing an earlier death rather than dealing with the underlying causes of their condition” (Pennings and Reid 2020). As well-known health journalist André Picard (2018) states, “vulnerable groups need to be simultaneously protected from exploitation and protected from exclusion. In other words, we need to find the balance between protecting them from harm and respecting their rights.” Similarly, Frazee (cited in CBC Radio 2020a) notes that MAiD legislation attempts to find a balance between autonomy and protecting the vulnerable, but she adds that autonomy has received disproportionate value and priority in this equation. In the following section, we discuss how autonomy was represented in the texts that we reviewed.

### Autonomy

The concept of autonomy received significant attention in the sample of news media texts we reviewed. Arguments in favor of MAiD often rest on the notion that access to this service provides suffering patients with the agency to end their lives on their own terms and exercise some control in a disempowering situation (Araujo 1994; Martin 2016). For instance, the plaintiffs in the Québec ruling (*Truchon v Canada*) that led to Bill C-7 argued that assisted dying should be a matter of personal choice, claiming that the reasonably foreseeable death criterion was paternalistic and restricted the choices of disabled people looking to end their suffering. Similarly, when it comes to restrictions for those with mental illness, Scully (2020) argues that there is a disconnect between the opinions of health experts and the lived expertise of those enduring complete and relentless mental despair. In other words, these advocates suggest that mentally ill people and people with disabilities should not be restricted from a choice that is available to others. Autonomy is depicted here as empowerment, as a leading value that grants patients some agency over their destiny.

However, the sources we reviewed revealed profound disagreement as to whether Bill C-7 promotes or undermines patient autonomy. In a piece titled “Liberty or Equality? Unrestricted Access to Medically Assisted Death Endangers Vulnerable People,” Enns (2019) highlights the tension between these competing values. She suggests that free choice regarding MAiD does not provide members of marginalized groups (such as those with disabilities or mental illnesses) the equality that they seek. She presents MAiD as a Hobson’s choice: a free choice in which only one thing is truly being offered (Enns 2019). More specifically, the argument suggests that since people with disabilities may face coercive and pressured environments, as well as power imbalances in the medical system, the option of MAiD cannot enable autonomous decision-making. The thinking is that, if people do not start out equal, removing safeguards will not equalize them (Enns 2019). In this instance, autonomy is presented as illusory or as a potential red herring that distracts us from the macro-level contexts in which decisions about MAiD are made.

Disagreements about MAiD and autonomy were also discussed within the prison context, where prisoners’ freedoms are already considerably constrained. Some inmate advocates, including the federal prison watchdog, expressed concerns about MAiD becoming a default option for those whose care choices are constrained in prison. Canadian prisons do not provide adequate palliative care, and they are not designed to support older and/or dying inmates. It is easier to access an assisted death than a compassionate or medical release from prison, and there is little political will to improve prisoners’ quality of life (Harris 2018). Harris (2018) urges for a compassionate parole system and a ban on MAiD in correctional facilities. However, others argue that a moratorium on MAiD in prisons removes choice and infantilizes inmates by assuming they cannot consent to the procedure (Mahboob 2020).

Lastly, several news pieces spoke about the coercive influence of poverty, homelessness, racialization, and limited social support services that may constrain people’s choices about end-of-life care (Dosani 2021; Harris 2020; CBC Radio 2020b). “Until people have a choice in how and where they live, it can never be a free choice on how to die” (Heidi Janz quoted in Boothby 2020). This framing often examines autonomy alongside dignity, which we discuss in the following section.

### Dignity

The concept of dignity also received considerable news media attention. Typically, dignity has been invoked in support of assisted dying in arguments about the importance of a dignified death (Dying with Dignity Canada 2021; Van Niekerk 2016; Reichstein 2019). The argument often contends that providing someone with the support to die peacefully, without pain, comfortably, and by their own choosing is more dignified than the alternative, which in some cases may be a far more unpleasant or unpredictable death (LeBlanc 2020). Although this language is still pervasive in MAiD-related discourse, we found a shift in recent news media pieces. About half of the articles that we reviewed invoke the concept of dignity in reference to the need to be able to live a dignified life (Peters 2020; CBC Radio 2020a). Generally, they contend that in the absence of the supports and services that permit people to live a dignified life, the option to “die with dignity” is concerning. Some articles suggest that MAiD may enable undignified living conditions by offering an easier remedy for suffering than the costlier alternative of providing supports (Ho, Favaro, and St. Philip 2020). In other words, it is more cost effective to provide people with MAiD than to expand the welfare state (i.e., social services) or provide costly end-of-life care.

One article included the story of 42-year-old Roger Foley who claimed that hospital staff offered him MAiD instead of providing him with the home care assistance he required, even though he made it clear that he wished to live at home (*CTV News *2018b). Foley was stuck in a hospital bed for over two years and publicized his audio recordings of hospital staff members attempting forced discharges with threats of hefty hospital bills and suggesting MAiD as a solution. In one audio recording:

[A] man is heard asking Foley how he’s doing and whether he feels like he wants to harm himself. Foley tells the man that he’s “always thinking I want to end my life” because of the way he’s being treated at the hospital and because his requests for self-directed care have been denied. The man is then heard telling Foley that he can “just apply to get an assisted [sic], if you want to end your life, like you know what I mean?” (*CTV News* 2018a).

Foley has insisted that he wants to live with dignity and that the solution for him is assisted life, not assisted death (*CTV News* 2018b). Despite the significance of that distinction, there is no evidence that his request for assisted life is taken as seriously as his potential eligibility for assisted death. Hence, these articles highlight a fear that assisted death will become “a cheaper option to providing quality community supports” (*CTV News* 2018a).

Other media pieces similarly note that MAiD has been considered as a potential solution for those struggling to obtain the supports needed to live a dignified life. One article reported on the hardships of people living on the Ontario Disability Support Program (ODSP). ODSP does not provide sufficient financial support to pay for food, medication, and rent—a problem further exacerbated by rising costs and the COVID-19 pandemic (Mulligan and Yawar 2020). Some ODSP recipients are left considering MAiD simply because they cannot afford to live (Mulligan and Yawar 2020; Peters 2020). As Frazee (quoted in CBC Radio 2020) notes, “it's cold comfort, I think, to be offered the choice to die when you are not offered the choice to live a dignified life.”

Canadian legal scholar Trudo Lemmens and Leah Krakowitz-Broker invoke the concept of dignity when speaking about Bill C-7. They argue that it will make dying with dignity easier than living with dignity. One portion of their argument specifically notes that the mandatory 90-day assessment period is an insufficient timeframe within which to determine if MAiD is the only possible solution to relieve a person’s suffering (Lemmens and Krakowitz-Broker 2020). Specifically, it may take longer than 90 days to access the necessary medical and financial supports. In 2019, Ontarians waited an average of 126 days to access specialized long-term care, and the application for Canada Pension Plan Disability Benefits takes approximately 120 days (Lemmens and Krakowitz-Broker 2020). Thus, they argue that a person can access MAiD and “die with dignity” long before they can access the supports needed to bolster their quality of life. Furthermore, in the case of a newly acquired disability or illness, it can take longer than 90 days for people to adjust to their new circumstances and envision what a dignified life will look like (Lemmens and Krakowitz-Broker 2020).

These media articles reflect a significant shift in the representation of dignity in the public discourse on MAiD. Rather than a primary focus on *dying* with dignity, recent conversations focus on the importance of ensuring that people can *live* dignified lives. More specifically, there is an ongoing debate about whether a dignified death via MAiD could be offered as a solution for those forced to live with the indignities of an inadequate welfare state. This tension has prompted further discussion about human rights violations, which we discuss below.

### Human rights

Human rights were represented as weighty moral considerations guaranteed by the state and appeared repeatedly in the media coverage. Although most scholars would concede that rights are not absolute, in that they can be overridden by other considerations (Nickel 2019), rights claims also hold high prioritization in a society’s moral framework. This positioning can make conflicting rights claims messy, with legal scholars often resorting to intricate weighting analyses for resolutions (Griffin 2008).

The media articles we reviewed reflect complex rights conflicts, often with stark disagreement about how people invoke rights claims. Some argue that a right to MAiD is already constitutionally guaranteed to citizens, while others argue that MAiD represents an infringement upon a citizen’s rights. The former argument posits that the legal right to life, liberty, and security of the person^4^ includes the right to make health and medical decisions, including those that will hasten death. Proponents contend that by preventing a Canadian from accessing MAiD, those citizens are compelled to seek less safe and unpredictable means of causing death, thus violating this *Charter* right (LeBlanc 2020). Some have referred to this as a “right to die” (Benatar 2010; Dying with Dignity Canada 2021). Other recent rights arguments described in the media contend that it is discriminatory to distinguish between mental and physical illnesses for the purposes of MAiD eligibility (Scully 2020). The argument here is that mental illness shares the same salient features of physical illness, and thus the “right to die” should be extended accordingly. More specifically, those with mental illnesses still suffer terrible pain for which there may be no options for relief. Hence, Scully (2020) argues that, to avoid unjust discrimination, the same argument for allowing MAiD should apply to the mentally ill so that they, too, are provided an option to avoid more gruesome or traumatic methods of suicide.

However, other texts represented countervailing rights claims (*CBC News* 2020; Lemmens and Krakowitz-Broker 2020; Boothby 2020), arguing that MAiD infringes on citizens’ rights. Most notably, this included an appeal to the right to equal protection and equal benefit of the law without discrimination. This argument contends that MAiD legislation represents a form of discrimination against those with disabilities and chronic illnesses (CBC Radio 2020a, 2020b). Lemmens and Krakowitz-Broker (2020) note that “the bill conforms to an ableist presumption that a life with disability or chronic illness is less worth living.” Lemmens and Krakowitz-Broker (2020) reiterate that Bill C-7 fails to explicitly require that all other reasonable options for those suffering with a disability or chronic illness be made available and tried before allowing MAiD. They present the concern that this system can prematurely facilitate the death of those individuals and constitutes a “deadly form of discrimination” against persons with disability or chronic illness (Lemmens and Krakowitz-Broker 2020).

Much of the current media discourse presents one or more of these conflicting rights claims, thus highlighting the chasm that exists between the opposing sides of this debate. These texts underscore the state’s role in upholding the rights of its citizens and indicate that the Government of Canada will need to determine how to strike the right balance between empowering autonomous decision-making and ensuring that people are protected from discrimination or undue influence.

## Discussion

As noted above, there is a strong focus on protecting the vulnerable in MAiD-related policy and news media texts. Our review highlights the extent to which concepts like *vulnerability* can be contextual. Vulnerability is not a static concept; it can change over time based on social, political, economic, and medical structures and circumstances. Our findings show that social determinants of health and social location influence perception regarding vulnerability, dignity, autonomy, and human rights (Dosani 2021; Frazee 2018). Individuals have the autonomy to make choices about their health and health care but not within conditions of their own choosing. Ableism, racism, and other forms of social marginalization are ever-present in our culture, economy, politics, and health care system. These structural forms of prejudice make particular groups and individuals more vulnerable to harm than others.

Several of the texts we reviewed highlight the importance of socioeconomic status (SES) in particular, which has received less attention in historical discussions about MAiD. There have long been discussions about MAiD from a disability rights perspective, and as such, it is not surprising that longstanding disability advocates had significant voices within the coverage we analyzed. Interestingly, more recent news media seems to be engaging with the fact that SES and poverty can have a significant impact on a) someone’s experience of being disabled and b) their feelings about the implications of Bill C-7.

As Frazee (2018) points out, the MAiD monitoring system does not sufficiently account for the extent to which poverty and insecure housing or income may inform requests to die. This is an area deserving of further public discourse. For instance, activist sb. smith has argued:

What these [MAiD advocates] don’t realize is that their class privilege clouds their perspectives on the way this legislation is and will continue to be used for harm with its money-saving potential to offload “burdens” (that is, disabled lives) from government-funded institutions like healthcare, housing, and so on. … It is increasingly necessary for financially privileged (and especially white, financially privileged) disabled people to confront and be forthcoming about their wealth and social status. This is especially important given that the community is overwhelmingly comprised of us crips who (barely) survive on government benefits or are altogether ineligible for said benefits, have multiple side hustles, are under-paid, are completely unable to work, live in subsidized and/or group housing, are under-housed, are routinely rejected from medical insurance coverage, often go without medical treatments and prescriptions, are often denied medical care, and so on. Rich disabled people do not know what it means to be viewed as financial “burdens” on the institution of the Canadian nation state. (smith 2021)

The socioeconomic disparity amongst people with disabilities seems especially pronounced when it comes to individuals such as Nicole Gladu, one of the plaintiffs in the Québec case that led to the creation of Bill C-7. Gladu has argued that her disability does not make her vulnerable, that she has traveled the world and lives independently in a 14th-floor condo with a beautiful view (Bryden 2020), and that she wants the right to die at a time of her choosing “with a flute of pink champagne in one hand and a canapé of foie gras in the other.”^5^ Gladu’s experience of life with a disability and her envisioned conclusion to it are radically different from those of Roger Foley, who feared being treated as a burden on the system (*CTV News* 2018a, 2018b). News articles such as the *National Post*’s piece titled “Euthanasia may save $139M, study says; Argues savings dwarf costs of assisted death” (Kirkey 2017) may reinforce this narrative.

It is also worth noting that in cases such as Foley’s, where it appears that health care providers are encouraging socioeconomically vulnerable patients towards MAiD, this may be a violation of the *Canadian Criminal Code*. The provision of information about MAiD is allowable under s. 241(5.1) and helps patients to make informed decisions about their end-of-life care. However, under s. 241(1), it is illegal to “counsel” someone to end their life:

“Counselling” for the purposes of s. 241(1) “concerns speech that, assessed objectively, aims to induce, persuade or convince a person to commit suicide.” … To counsel a patient to die by suicide remains illegal. For the purposes of the *Criminal Code*, “to counsel” means to provide information with the aim of inducing, persuading, or convincing a person to die by suicide. (CAMAP 2022, 2)

Those in the media reporting on stories like Foley’s would do well to examine and clarify this distinction. News media stories could use further nuance to emphasize the responsibility of health care workers to uphold the law, respect the existing safeguards, and honor patients’ dignity and human rights.

Given the growing concern that MAiD is more readily available than some of the supports necessary for a dignified life, it is unsurprising that news media about MAiD increasingly focuses on the role of government. The state is responsible for upholding welfare, safety, security, and human rights (Stone 2012). Though there are ideological debates about the appropriate degree and methods of state intervention in people’s lives, Canadian law and the Canadian *Charter of Rights and Freedoms* recognize these fundamental state responsibilities. Governments can produce, sustain, and ameliorate health inequities. Given the steps Canada has taken to enable dignified deaths through the availability of MAiD, it should bear the counterbalancing responsibility to ensure that dignified lives are enabled through adequate supports, including disability, income, housing, and mental health supports as well as medical, home, and palliative care.

Policymakers will especially need to account for the impacts that MAiD can have on individuals who are struggling with poverty and unmet needs. Some jurisdictions and regulatory bodies have attempted to address this by requiring that healthcare practitioners seek to understand a patient’s circumstances, perspective, and reason for contemplating MAiD (CPSA 2019) and query whether the expressed request is transient or an expression of suffering due to unmet needs that can be alleviated or addressed by other means (CNA 2017). For patients for whom death is not reasonably foreseeable, the “MAID provider must ensure the patient has been informed of the means available to relieve their suffering, including, where appropriate: counseling services, mental health and disability support services, community services and palliative care and has been offered consultations with relevant professionals who provide those services or that care” (CPSO 2021). However, existing policies and legislation do not address how exactly healthcare professionals should assess for the social determinants of health or what exactly they should do when MAiD requests are made as a result of unmet needs, poverty, or lack of access to supports.

The fear that Bill C-7’s permissiveness will negatively impact individuals with disabilities or who are struggling financially appears to be bearing out in recent news stories. In the months following our news media review, several cases have received national attention in which individuals requested and/or received MAiD due to a lack of finances, health care, and/or supports. In one instance, Donna Duncan, a British Columbia woman suffering with post-concussion syndrome and mental health issues, was unable to access medical care for an extended period of time during the COVID-19 pandemic. Duncan surprised her family by revealing that she had applied for MAiD, been assessed, and been approved for the procedure in the span of a few days (Daflos 2022). Given the complexity of her case and the fact that her family believed that her mental health issues had compromised her capacity to make this decision, there was a police inquiry into her assisted death. In another instance, Tracey Thompson, a Toronto resident, had been enduring severe long-COVID illness for two years. With an absence of medical care to treat her condition, she was rendered unable to work. After over two years of lost income and without access to provincial funding for individuals living with disabilities, she began the process of applying for MAiD. She noted that “[MAiD] is exclusively a financial consideration,” adding that her “choices are basically to die slowly and painfully, or quickly” (Tracey Thompson quoted in Alberga 2022). Lastly, a 51-year-old woman from Ontario, identified as Sophia, was living with the chronic illness Multiple Chemical Sensitivities (MCS) and applied for MAiD because she could not access affordable housing free of chemical cleaners and cigarette smoke (Favaro 2022). Despite the advocacy efforts of a team of physicians to provide her with alternative living conditions, Sophia received MAiD. In a video filmed about a week before her death, she claimed, “the government sees me as expendable trash, a complainer, useless and a pain in the ass” (Sophia quoted in Favaro 2022). Several news articles that we reviewed expressed concerns that the current legislative and policy environment could enable access to assisted death more readily than access to a dignified life. These most recent news stories demonstrate that such a fear is not unfounded and remains a critical area for research and policy discussion.

Although Bills C-14 and C-7 can always be amended and improved upon, they exist in a fundamentally unequal and inequitable society. MAiD policy can be revised to better address social and health inequities in its application. However, solving socioeconomic issues and addressing the social determinants of health require work beyond the scope of MAiD legislation and policy. Many of the articles we reviewed suggested that the state should have a more comprehensive approach to address structural, social, and economic inequities.

## Conclusion

The dissensus that our media analysis uncovered cannot be easily resolved. Though key stakeholders appear to share the values of autonomy, dignity, and human rights, they appeal to them in diverse ways and with sometimes conflicting policy demands. A discursive approach to a corporeal procedure like MAiD is valuable because the stories we analyze function pedagogically. People learn who they are and what it means to have a full dignified life from myriad sources, and those include news media. Thus, our findings about the concerns, priorities, and experiences of key stakeholders are also findings about what an average reader of mainstream news in Canada can learn about not only who is vulnerable but how they become assigned to that category and what it means to be considered vulnerable. Those lessons affect ideas about who can and should have autonomy. They alter considerations of dignity, reinserting the term with significance when it is yoked to life rather than death. They prompt assessment of human rights in relation to accessibility. They offer a useful gauge of how public and stakeholder views on MAiD continue to shift alongside changes in federal legislation and have the potential to change national conversations.
